# Enhancing Mental Health Support for Unaccompanied Asylum- Seeking Young People (UASYP): A Collaborative Pathway in Aberdeen

**DOI:** 10.1192/bjo.2025.10497

**Published:** 2025-06-20

**Authors:** Praveen Kumar, Claire Hardie

**Affiliations:** City Hospital, Aberdeen, United Kingdom

## Abstract

**Aims:** This initiative aimed to establish a structured and collaborative pathway to address the mental health and psychosocial needs of Unaccompanied Asylum-Seeking Young People (UASYP) in Aberdeen. The project sought to provide trauma-informed, culturally sensitive interventions while fostering partnerships with local and national agencies to ensure comprehensive support. In 2023, the UK received 3,412 applications from Unaccompanied Asylum-Seeking Children (UASC), with Scotland accommodating a proportionate share through the National Transfer Scheme.

**Methods:** The pathway was developed within the Child and Adolescent Mental Health Services (CAMHS) at City Links Hospital, Aberdeen, inspired by Professor Renos Papadopoulos’ frameworks on refugee trauma and the “Enhancing Vulnerable Asylum Seekers’ Protection” handbook. Referrals were limited to UASYP with looked-after status, ensuring targeted support for the most vulnerable. Initial network meetings involved key stakeholders, including social workers, guardians, and lawyers, to assess the young person’s needs and determine appropriate interventions. Consent processes were designed to facilitate transparent communication between stakeholders and ensure ethical information-sharing. Collaborations with agencies like Aberlour, the Anchor Unit, and Freedom from Torture were integral. Data from 13 cases were analyzed to evaluate demographic trends, service engagement, and outcomes.

**Results:** The cohort had an average age of 17.2 years, representing countries including Afghanistan, Iran, Eritrea, and Sudan. Language barriers were notable, with Pashto, Tigrinya, and Kurdish Sorani as primary languages. Only 5% of referrals progressed to CAMHS, underscoring the selective nature of the pathway. Most referrals resulted in external partnerships, particularly with organizations like Freedom from Torture, or redirection to community resources. Guardians, managed through Aberlour in collaboration with the Anchor Unit, played a pivotal role as stable third parties, addressing the power dynamics inherent in social worker relationships.

**Conclusion:** This pathway highlights the value of integrating trauma-informed care with a networked, multidisciplinary approach to support UASYP. By leveraging existing frameworks and fostering agency partnerships, the initiative demonstrated the feasibility of providing culturally sensitive care tailored to the unique needs of asylum-seeking young people. Given the increasing number of UASC arrivals in the UK, further development of Tier 2 group interventions and ongoing evaluation of pathway outcomes are recommended to expand the model’s impact.

